# Supplementary material for: Unraveling the influence of defects on Sulfonamide adsorption onto Blue-phosphorene nanotube using density functional theory
Source: PLoS One. 2025 Mar 24;20(3):e0312034. doi: 10.1371/journal.pone.0312034 (PMC11932492; doi:10.1371/journal.pone.0312034)
Supplement: S1 Text — (PDF) [file pone.0312034.s001.pdf]

Supplementary Material: Unraveling the influence of defects on Sulfonamide adsorption onto Blue-phosphorene nanotube using density functional theory

J. M. Vergara[a], J. D. Correa[a], M.E. Mora-Ramos[b], E. Flórez[a]

[a] *Facultad de Ciencias Básicas, Universidad de Medellín, Medellín, Colombia*
[b] *Centro de Investigación en Ciencias, Instituto de Investigación en Ciencias Básicas y Aplicadas, Universidad Autónoma del Estado de Morelos, CP 62209, Cuernavaca, Morelos, México*

Preprint submitted to Elsevier *September 11, 2024*

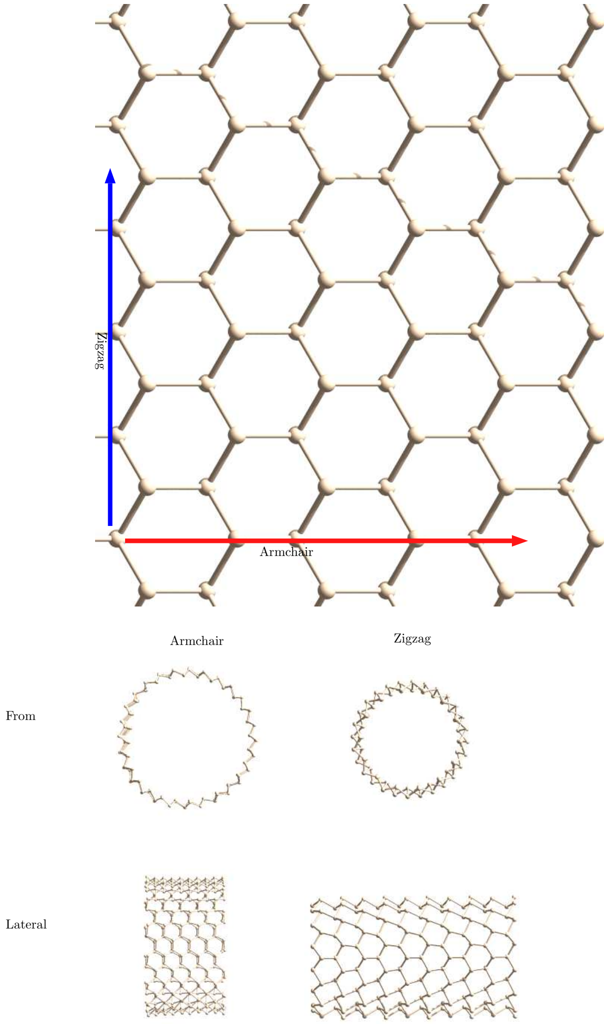

Figure S1: Geometric representation of the Blue Phosphorene Monolayer. The red vector marks the armchair symmetric direction, and the blue vector marks the zigzag symmetry direction. Lateral and from the view of the resultant ZZ and AM, blue phosphorene nanotubes are included.

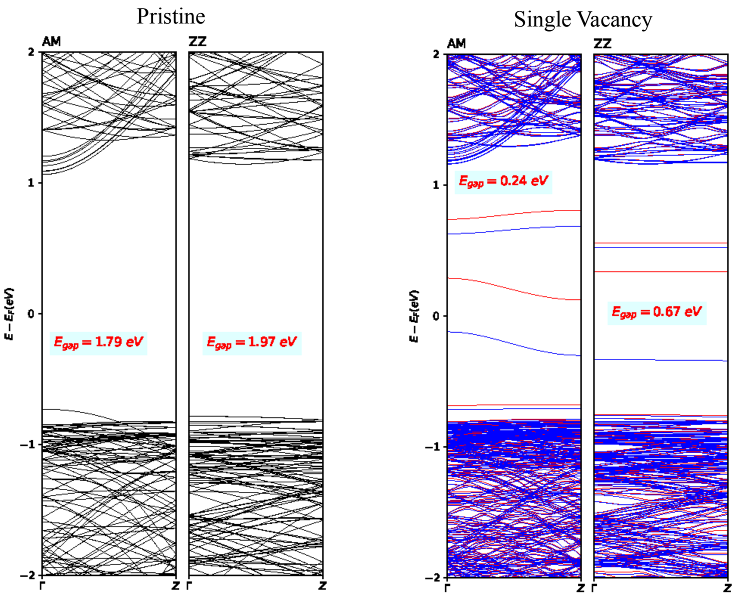

Figure S2: Band structure of pristine and single vacancy armchair and zigzag blue phosphorene nanotube.

**System**	**SAM position**	E_{ads} **(without VdW)**	E_{ads} **(with VdW)**
Pristine ZZ	**Benzene**	**0,174**	**-0,279**
	NH_2	-0,030	-0,191
	SO_2NH_2	-0,008	-0,130
	$2H$	0,197	-0,094
Pristine AM	**Benzene**	**0,199**	**-0,312**
	$2H$	0,131	-0,205
	NH_2	0,156	-0,053
	SO_2NH_2	0,136	-0,028
SV ZZ	**Benzene**	**0,168**	**-0,517**
	$2H$	0,035	-0,497
	NH_2	-0,033	-0,380
	SO_2NH_2	0,048	-0,272
SV AM	**Benzene**	**0,226**	**-0,373**
	$2H$	0,249	-0,221
	NH_2	0,094	-0,161
	SO_2NH_2	0,173	-0,122

Table S1: Results interaction of pristine and single-vacancy (SV) with SAM molecules with and without taking into account Van der Wals interactions; Adsorption energy (E_{ads}) is presented in eV.

**Molecule**	**Position**	**System**	E_{ads} **without VdW corrections**
**SDZ**	Benzene	Pristine ZZ	0,087
		Pristine AM	0,120
**SMD**	Benzene	Pristine ZZ	0,050
		Pristine AM	0,173
**SMR**	Benzene	Pristine ZZ	0,239
		Pristine AM	0,241
**SMT**	Benzene	Pristine ZZ	0,135
		Pristine AM	0,222
**SMX**	Benzene	Pristine ZZ	0,258
		Pristine AM	0,094

Table S2: Results interaction of pristine with different Sulfonamides molecules without taking into account Van der Wals interactions; Adsorption energy (E_{ads}) is presented in eV.

			$\theta_0 = 10^{12}$ (Visible light)			
**System**	**Molecule**	**Position**	τ $300K$	τ $310K$	τ $320K$	τ $330K$
Pristine ZZ	**SAM**	**Benzene**	$4,9 \times 10^{-08}$	$3,4 \times 10^{-08}$	$2,5 \times 10^{-08}$	$1,8 \times 10^{-08}$
		NH_2	$1,6 \times 10^{-09}$	$1,3 \times 10^{-09}$	$1,0 \times 10^{-09}$	$8,3 \times 10^{-10}$
		SO_2NH_2	$1,5 \times 10^{-10}$	$1,3 \times 10^{-10}$	$1,1 \times 10^{-10}$	$9,7 \times 10^{-11}$
		$2H$	$3,8 \times 10^{-11}$	$3,4 \times 10^{-11}$	$3,0 \times 10^{-11}$	$2,7 \times 10^{-11}$
Pristine AM	**SAM**	**Benzene**	$1,8 \times 10^{-07}$	$1,2 \times 10^{-07}$	$8,3 \times 10^{-08}$	$5,9 \times 10^{-08}$
		$2H$	$2,8 \times 10^{-09}$	$2,2 \times 10^{-09}$	$1,7 \times 10^{-09}$	$1,4 \times 10^{-09}$
		NH_2	$7,9 \times 10^{-12}$	$7,4 \times 10^{-12}$	$6,9 \times 10^{-12}$	$6,5 \times 10^{-12}$
		SO_2NH_2	$2,9 \times 10^{-12}$	$2,8 \times 10^{-12}$	$2,7 \times 10^{-12}$	$2,6 \times 10^{-12}$
SV ZZ	**SAM**	**Benzene**	$4,8 \times 10^{-04}$	$2,5 \times 10^{-04}$	$1,4 \times 10^{-04}$	$7,8 \times 10^{-05}$
		$2H$	$2,2 \times 10^{-04}$	$1,2 \times 10^{-04}$	$6,7 \times 10^{-05}$	$3,9 \times 10^{-05}$
		NH_2	$2,4 \times 10^{-06}$	$1,5 \times 10^{-06}$	$9,7 \times 10^{-07}$	$6,4 \times 10^{-07}$
		SO_2NH_2	$3,7 \times 10^{-08}$	$2,6 \times 10^{-08}$	$1,9 \times 10^{-08}$	$1,4 \times 10^{-08}$
SV AM	**SAM**	**Benzene**	$1,9 \times 10^{-06}$	$1,2 \times 10^{-06}$	$7,6 \times 10^{-07}$	$5,0 \times 10^{-07}$
		$2H$	$5,1 \times 10^{-09}$	$3,9 \times 10^{-09}$	$3,0 \times 10^{-09}$	$2,4 \times 10^{-09}$
		NH_2	$5,0 \times 10^{-10}$	$4,1 \times 10^{-10}$	$3,4 \times 10^{-10}$	$2,8 \times 10^{-10}$
		SO_2NH_2	$1,1 \times 10^{-10}$	$9,7 \times 10^{-11}$	$8,4 \times 10^{-11}$	$7,4 \times 10^{-11}$

			$\theta_0 = 10^{16}$ (UV light)			
**System**	**Molecule**	**Position**	τ $300K$	τ $310K$	τ $320K$	τ $330K$
Pristine ZZ	SAM	**Benzene**	$4,9 \times 10^{-12}$	$3,4 \times 10^{-12}$	$2,5 \times 10^{-12}$	$1,8 \times 10^{-12}$
		NH_2	$1,6 \times 10^{-13}$	$1,3 \times 10^{-13}$	$1,0 \times 10^{-13}$	$8,3 \times 10^{-14}$
		SO_2NH_2	$1,5 \times 10^{-14}$	$1,3 \times 10^{-14}$	$1,1 \times 10^{-14}$	$9,7 \times 10^{-15}$
		$2H$	$3,8 \times 10^{-15}$	$3,4 \times 10^{-15}$	$3,0 \times 10^{-15}$	$2,7 \times 10^{-15}$
Pristine AM	SAM	**Benzene**	$1,8 \times 10^{-11}$	$1,2 \times 10^{-11}$	$8,3 \times 10^{-12}$	$5,9 \times 10^{-12}$
		$2H$	$2,8 \times 10^{-13}$	$2,2 \times 10^{-13}$	$1,7 \times 10^{-13}$	$1,4 \times 10^{-13}$
		NH_2	$7,9 \times 10^{-16}$	$7,4 \times 10^{-16}$	$6,9 \times 10^{-16}$	$6,5 \times 10^{-16}$
		SO_2NH_2	$2,9 \times 10^{-16}$	$2,8 \times 10^{-16}$	$2,7 \times 10^{-16}$	$2,6 \times 10^{-16}$
SV ZZ	SAM	**Benzene**	$4,8 \times 10^{-08}$	$2,5 \times 10^{-08}$	$1,4 \times 10^{-08}$	$7,8 \times 10^{-09}$
		$2H$	$2,2 \times 10^{-08}$	$1,2 \times 10^{-08}$	$6,7 \times 10^{-09}$	$3,9 \times 10^{-09}$
		NH_2	$2,4 \times 10^{-10}$	$1,5 \times 10^{-10}$	$9,7 \times 10^{-11}$	$6,4 \times 10^{-11}$
		SO_2NH_2	$3,7 \times 10^{-12}$	$2,6 \times 10^{-12}$	$1,9 \times 10^{-12}$	$1,4 \times 10^{-12}$
SV AM	SAM	**Benzene**	$1,9 \times 10^{-10}$	$1,2 \times 10^{-10}$	$7,6 \times 10^{-11}$	$5,0 \times 10^{-11}$
		$2H$	$5,1 \times 10^{-13}$	$3,9 \times 10^{-13}$	$3,0 \times 10^{-13}$	$2,4 \times 10^{-13}$
		NH_2	$5,0 \times 10^{-14}$	$4,1 \times 10^{-14}$	$3,4 \times 10^{-14}$	$2,8 \times 10^{-14}$
		SO_2NH_2	$1,1 \times 10^{-14}$	$9,7 \times 10^{-15}$	$8,4 \times 10^{-15}$	$7,4 \times 10^{-15}$

Table S3: Results interaction of pristine and single-vacancy (SV) with SAM molecule; recovery time (τ) is presented in s.
